# Identification and weighting of kidney allocation criteria: a novel multi-expert fuzzy method

**DOI:** 10.1186/s12911-019-0892-y

**Published:** 2019-09-06

**Authors:** Nasrin Taherkhani, Mohammad Mehdi Sepehri, Shadi Shafaghi, Toktam Khatibi

**Affiliations:** 1 Group of Information Technology, Faculty of Industrial and Systems Engineering, Tarbiat Modares University, Tehran, 1411713116 Iran; 2 Faculty of Industrial and Systems Engineering, Tarbiat Modares University, Tehran, 1411713116 Iran; 3 Lung Transplantation Research Center, National Research Institute of Tuberculosis and Lung Diseases (NRITLDD), Shahid Beheshti University of Medical Sciences, Tehran, Iran

**Keywords:** Kidney allocation, Fuzzy Delphi method, Intuitionistic fuzzy AHP, Kidney allocation criteria

## Abstract

**Background:**

Kidney allocation is a multi-criteria and complex decision-making problem, which should also consider ethical issues in addition to the medical aspects. Leading countries in this field use a point scoring system to allocate kidneys. Hence, the purpose of this study is to identify and weight the kidney allocation criteria considering the balance between utility and equity.

**Methods:**

To do this, a new fuzzy hybrid approach is proposed, which consists of two steps: In the first step, Fuzzy Delphi Method (FDM) is used to identify the effective criteria in the kidney allocation algorithm. In the second step, Intuitionistic Fuzzy Analytic Hierarchy Process (IF-AHP) is employed to determine the weight of the criteria.

**Results:**

The results showed that the highest weight belongs to “Medical emergency” criterion and the lowest weight to “5 HLA mismatches”, which is similar to Euro-transplant kidney allocation system (ETKAS). The developed method is evaluated in two steps. First, the proposed model is implemented using a real case study from the Iranian Kidney Allocation System. It was shown that the proposed model has the potential to improve allocation outcome. Second, the proposed model’s superiority to the current model is approved by the experts using the results display in the profile matrix. Finally, sensitivity analysis is performed to check the robustness of the proposed model.

**Conclusions:**

This paper contributes to the kidney allocation literature by doing the following: (a) developing a comprehensive framework for identification and weightings of criteria for kidney allocation, (b) using, for the first time, the IF-AHP technique to consider hesitancy of decision makers and uncertainty in organ allocation, and (c) proposing an appropriate framework for the countries that intend to improve or modify their organ allocation system.

**Electronic supplementary material:**

The online version of this article (10.1186/s12911-019-0892-y) contains supplementary material, which is available to authorized users.

## Background

In recent years, medical knowledge has paid special attention to organ transplantation so that the concerns of patients in the end stages of failure of the organ have been improved significantly. Among the organs that can be transplanted, the kidneys have the highest demand. In the United States, as of September 2018, there were about 114,000 patients on the waiting list of organ transplantation, with more than 94,000 of them requiring kidney transplantation (82.45%) [1]. In Iran, as of October 2018, 80.34% of the candidates’ waiting lists required kidney transplant [2]. Among the most important challenges in organ transplantation process are the organ allocation policies and finding the most appropriate recipient. Over time, organ allocation policies have changed and improved. Stegall et al. have explained the changes and modifications that have occurred in the kidney allocation system in the United States from 2004 to 2014, leading to the new Kidney Allocation System (KAS) [3]. The central concept is that kidney allocation should create a balance between utility (the best use of kidneys) and equity (equal access to kidneys for all wait-listed patients). Currently, many allocation policies have been developed with the aim of balancing between utility and equity.

There is a lot of fundamental debate about balancing between the utility and equity criteria. When the sub-criteria of equity such as medical urgency are used to rank patients, some decision makers may have this question in mind: “Is it ethical to differentiate between patients due to their medical conditions?” [4]. Conversely, when utility sub-criteria are considered, others may ask: “Is it ethical to distinguish between patients who will have more survival than those who survive less?” [5]. Of course, there are researches with no clear-cut resolution to this issue, and their goal is to solely find a balance between utility and equity [6].

There are many organizations in the world for organ transplantation such as United Network for Organ Sharing (UNOS) in the United States, Euro-transplant in Europe, and Organization Nacional de Transplant (ONT) in Spain. In Iran, there is currently no comprehensive and integrated system for organ transplantation. Of course, efforts have been made to create an integrated network for organ procurement and transplantation. Iranian Network for Organ Procurement and Transplantation (IRNOPT) is the result of this effort [7]; however, it has not been implemented yet.

According to the geographical extent of Iran, the different regions of the country perform locally the process of organ procurement and allocation. In the case of kidney, the country is divided into 15 regions, and each region has its own waiting list, but the same algorithm used in each of them. If an organ is available in one region, allocation is done locally. It rarely happens that the kidneys are sent from one region to another. Any patient needing kidney transplantation has to register in the waiting list of a region (a patient may register at the same time in the list of several regions). When a donation kidney is available, the allocator (an expert person who is responsible for organ allocation) tries to select the most suitable candidate by searching in the waiting list. To select the most suitable candidate, the waiting list is filtered based on the blood type and medical urgency. Then she/he sorts the list based on the waiting time, and determines six high priorities based on the two factors: 1. The distance between the patient’s place of residence and the TC (can the recipient reach the TC at the right time?) 2. Age difference between the donor and the recipient (is the age difference between the donor and the recipient appropriate?). Two high priorities are kidney recipients and four next priorities are in reservation mode. For any reason, if two high priorities cannot receive the kidney, the other four booked priorities are prepared for transplantation. In fact, the existing method is largely equity-based and does not pay much attention to the criteria that affect the graft survival. Although the age difference is one of the factors that increases utility, but in existing method it is not the key factor for allocation. Only in the final step, the expert checks that the age difference is not outside the specified range (less than 15 years). However, the allocator tries to make the best selection by taking all the criteria into consideration. Given that the waiting list is gradually reduced by filtering, and finally, the expert selects the recipients among the remained candidate in the filter list, the overall effect of all criteria is not considered. On the other hand, the likelihood of human error and emotions can affect the outcome of the allocation. Hence, there are several reasons to change and modify the current allocation algorithm. The most important reason for changing the current system is to improve the overall survival of the transplant and patients. A system that allocates kidneys based on utility criteria would reduce the number of re-transplantation or delay it, and slow down the growth of the waiting list [8]. With this view, Stegall proposed a method for allocating “The right kidney for the right recipient” [9].

Leading countries in this field use a point scoring system to allocate kidneys. In these systems, different points are considered for each criterion. The donated kidney is allocated to the patient who earns the highest score. The most important challenge in designing these systems is identifying effective factors and weighting them to get the best utility and equity outcomes. Another problem is to consider the decision makers’ hesitation and uncertainly in comparing the factors for weighting them. Therefore, the purpose of this study is to identify and weight the kidney allocation criteria considering the balance between utility and equity. It reviews current kidney allocation algorithms and proposes a new integrated two-step framework for developing kidney allocation algorithm. First, Fuzzy Delphi Method (FDM) is used to identify the criteria in the kidney allocation algorithm. Next, Intuitionistic Fuzzy Analytic Hierarchy Process (IF-AHP) is employed to determine the weight of the criteria considering the hesitation and uncertainly of decision makers in their decisions.

### Research background

A lot of research has been done to improve the allocation algorithm from different aspects. David and Yechiali provided a model for allocating multiple organs to multiple recipients. The purpose of their study was to develop an allocation model, which optimizes various criteria [10]. Yuan et al. developed a kidney allocation fuzzy expert system to assist medical doctors in situations where they deal with ambiguity and complexity. They showed that the fuzzy logic-based policy is very close to an expert’s opinion [11]. Gundogar et al. developed the fuzzy organ allocation system (FORAS) for patients requiring kidney transplantation. FORAS determines which patients should receive a kidney when it becomes available. They used a simulation to show that FORAS is more useful than other kidney allocation systems [12]. Baskin & Nyberg designed a utility-based system to balance the supply and demand of kidney transplantation. They sought to maximize the total number of years of kidney allograft function using the recipient risk score and the deceased donor score [8]. In another study, Cruz-Ramirez et al. proposed a rule-based decision making system to allocate the liver [13]. Bertsimas et al. used a linear regression to determine the weight of the score elements. They focused on national allocation policies in the US. They found acceptable score weights by solving the optimization problem [14]. Tong et al. proposed a method for kidney allocation based on patient’s preference [15]. Al-Ebbini et al. developed a fuzzy lung allocation system (FLAS) to show that systems designed with fuzzy logic are closer to the expert’ opinion than other systems [16]. Ahmadvand and Pishvaee developed a model based on Data Envelopment Analysis (DEA) for kidney allocation. Their objective was to find the best patient-organ pairs to increase the fitness of kidney allocation in conditions of uncertainty [17]. Scalia et al. used Delphi method to identify and determine the importance of factors that can affect the pancreatic islet transplant outcomes. The quantitative criteria were evaluated with crisp value (crisp is used in contrast with fuzzy. A crisp number has a precise value but a fuzzy number has a possible range of values) and the qualitative criteria with linguistic scales [18]. Dongping et al. identified long-term factors influencing survival after kidney transplantation with Delphi method in three rounds [19]. Nosotti et al. used a modified Delphi technique to identify the criteria for recipient selection [20].

The application of AHP to organ transplantation was studied in the 1990s. Cook et al. used AHP to develop a ranking system for allocation of cadaver liver. The criteria considered were waiting time, logistics, tissue compatibility, financial situations and medical status [21]. Koch designed an AHP model that included the medical and social criteria. He concluded that the effect of social criteria should not be ignored [22]. Another research by Koch & Rowell considered quantitative and qualitative criteria using AHP technique to allocate an organ. The criteria included: survival, intelligence, social recognition, physical independence, compliance and activity following a successful transplant [23, 24]. Saha et al. developed a knowledge-based system to select the optimal donor-recipient by applying fuzzy techniques and AHP. They used a Mamdani Style Fuzzy Inference System (MSFIS) to select potential donor-recipient candidates. Then AHP was applied to rank the recipient for a donated kidney. The criteria included: selection, matching, location and transplant status [25]. Lin et al. proposed a multi-criterion decision-making model using AHP for liver allocation. They considered four criteria of benefit, efficiency, equity and urgency [26].

Based on the past research, it is clear that AHP is a useful tool for solving an organ allocation problem; however, for dealing with the uncertainty expressed in the expert opinions, fuzzy sets are suitable tools. Therefore, it is necessary to move the AHP model with crisp numbers to the fuzzy one [27]. Fuzzy AHP has been extensively used in recent research works, especially in the healthcare area [28–31].

### Research gap

Most of the existing literature discusses the allocation process and develops the system for allocation without clearly developing an integrated framework from the beginning that includes identification and weighting of the effective criteria in the organ allocation, similar to what is presented in this research. The detailed literature analysis shows that the Delphi and AHP methods are common methods in the development of organ allocation systems, but they have been used separately in the literature. To the best of our knowledge, there is no study, which integrates Delphi with AHP together in developing organ allocation system and fills the gap in the literature. On the other hand, in the traditional AHP, uncertainty of the decision maker is not formulated in the pairwise comparison of criteria. Traditional AHP uses a linguistic scale whose numerical values are between 1 and 9. A linguistic evaluation such as “very strong importance” is represented by 7 in the scale of traditional AHP. However, the decision maker’s judgment “very strong importance” may not be clear enough to assign ‘7’. His/her opinion might mean around 7. Fuzzy sets are excellent tools for dealing with such type of uncertainties. Furthermore, the intuitionistic fuzzy sets can reflect the hesitancy of decision makers whereas classical sets cannot deal with it [32]. In situations where uncertainty is high to have a reliable analysis, using intuitionistic fuzzy numbers instead of fuzzy number is better [27]. Intuitionistic Fuzzy AHP is extensively used in the current literature [27, 32–35], but in the organ allocation literature, we observed no study that used the IF-AHP technique. In this study, IF-AHP technique has been used to weigh the criteria.

## Methods

**Fig. 1:**
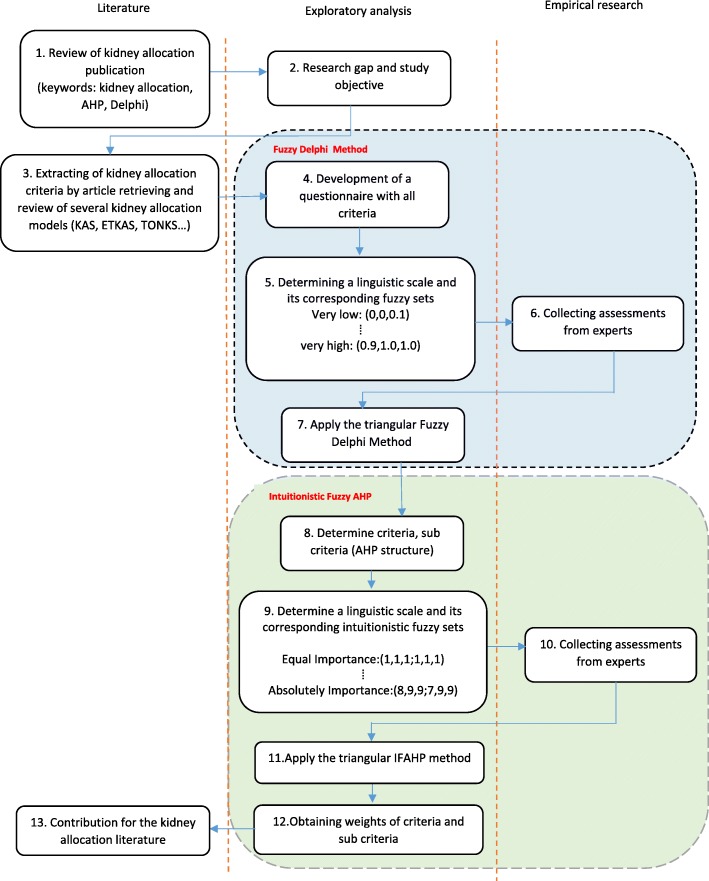
Proposed framework for identifying and weighting the essential criteria of kidney allocation

In this section, the proposed methodology as well as the tools used in each of the steps are explained and discussed. The purpose of this study is to identify and weight the kidney allocation criteria. In order to achieve this goal, the research was carried out in two main steps. In the first step, a questionnaire was developed based on the criteria extracted from the literature and different allocation systems, the first step questionnaire was designed, which is available as Additional file 1. The questionnaires were answered by 10 experts, who were mainly decision makers and policy makers in organ allocation in Iran. Since the questionnaires were completed face-to-face, all questionnaires were answered. There are different opinions about the size of the panel in Delphi method. According to Kardaras et al., different researchers have proposed a range of 5 to 31 experts [36]. Therefore, the panel size considered in this study is appropriate. After collecting the questionnaires, we identified the essential factors in kidney allocation using FDM. The goal of the second step was to weight the criteria identified in the first step to prioritize patients in the waiting list. IF-AHP was used to determine the weight of the criteria. For the second step, based on the identified factors, another questionnaire was designed to weigh the factors by IF-AHP method, which is available as Additional file 2. The second questionnaire was completed by the same respondents. The flowchart of the proposed methodology is shown in Fig. 1.

To evaluate the proposed method, we used the kidney transplantation dataset in Tehran from October 2017 to December 2017. This dataset includes information about both the patients registered on the kidney waiting list (484 registered patients) and deceased donors (124 donors). It is notable that there was no factor that indicates the outcomes of the transplant, such as the graft survival in the dataset. Therefore, we used the Estimated Post Transplant Survival (EPTS) score to evaluate the transplantation results and compare it with the proposed model.

EPTS score is a numerical measure used in the new kidney allocation system in UNOS. This is a percentage score that ranges from 0 to 100%. Candidates with a lower EPTS score are expected to have more graft survival compared to those with higher EPTS score. We used the EPTS calculator, provided by UNOS, to calculate the EPTS score. See for more details in [37].

### Step 1: identification of kidney allocation factors with FDM

The Delphi technique was developed by Dalkey and Helmer at the Rand Corporation [38]. Since then, it has been used extensively in many areas. The purpose of this method is to acquire the most reliable consensus of a group of experts’ opinion. In many real situations, expert’ judgment cannot be expressed with crisp numbers, and using linguistic scales is more commonplace and more convenient for experts [39]. To overcome these problems, this study uses FDM.

FDM is a combination of fuzzy set theory (proposed by Zadeh in 1965 [40]) and Delphi method. Ishikawa developed FDM with triangular fuzzy numbers in 1993 [41].

**Fig. 2:**
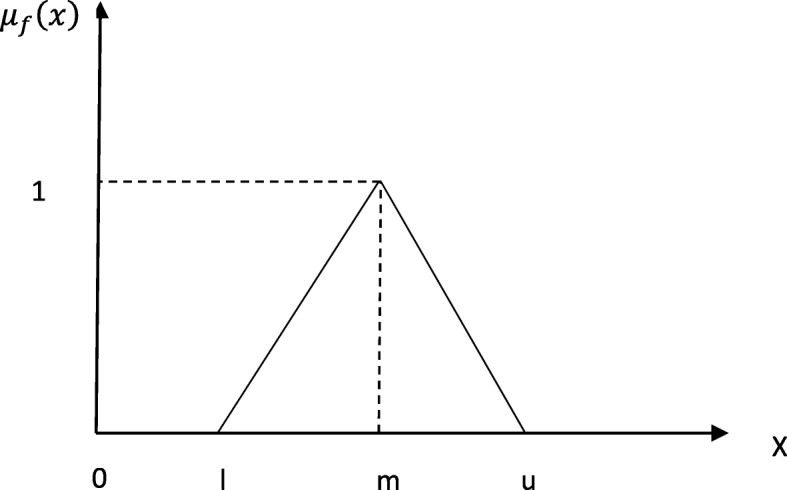
Membership functions of TFN

Here, the required information is collected in the form of linguistic scales from experts and analyzed by fuzzy method. This study uses fuzzy triangular numbers to convert linguistic scales to fuzzy numbers (Table 1). Each fuzzy triangular number is displayed with three real numbers F = (l, m, u) in which l is the minimum value of F, u is the maximum value of F, and m is the most probable value of fuzzy number [42] (see Fig. 2). Membership function of a triangular fuzzy number is as follows:

1 $$ {\mu}_f(x)=\left\{\begin{array}{c}\frac{x-l}{m-l}\ l\le x\le m\\ {}\frac{u-x}{\mathrm{u}-m}\ m\le x\le u\\ {}0\  othewise\end{array}\ \right. $$

**Table 1:** shows how to define linguistic scales in triangular fuzzy numbers [42]

Linguistic Scales	Fuzzy number
Very low	(0, 0, 0.1)
Low	(0, 0.1, 0.3)
Medium low	(0.1, 0.3, 0.5)
Medium	(0.3, 0.5, 0.7)
Medium high	(0.5, 0.7, 0.9)
High	(0.7, 0.9, 1.0)
Very high	(0.9, 1.0, 1.0)

After the experts’ opinions are collected and fuzzified (turned into fuzzy numbers), the opinions should be aggregated. Different methods have been proposed for aggregation of experts’ opinions. This study uses a geometric mean model that has been used in many studies [43–46].

Assume the fuzzy number $\tilde{a}_{i}j$ to be the *j*^*th*^ factor importance of the *i*^*th*^ expert and it is given as follows: $\tilde{a}_{i}j=\left({a}_{ij}.{b}_{ij}.{c}_{ij}\right)$ for i = 1,2,3, …,n; j = 1,2,3, …,m. To aggregate the judgments of all experts, Eq. (2) will be used:

2 $$ \tilde{a}_{j}=\left(\mathit{\min}\left\{{a}_{ij}\right\}.{\left(\prod \limits_{i=1}^n{b}_{ij}\right)}^{\raisebox{1ex}{$1$}\!\left/ \!\raisebox{-1ex}{$n$}\right.}.\mathit{\max}\left\{{c}_{ij}\right\}\right) $$

Finally, to determine the importance of criteria, the aggregated result is better to be transformed into a crisp value, and then compared with the threshold (a). In this study, we used a center of the gravity method to defuzzify the fuzzy values [42].

The value of threshold is calculated by determining the average of all factors’ weight:

- If *a*_*j*_ ≥ *a* , then factor j is selected.
- If *a*_*j*_ < *a* , then factor j is rejected.

### Kidney allocation criteria

In this section, the most important criteria for kidney allocation are described briefly. These criteria have been extracted by reviewing the literature and different allocation systems currently implemented in other parts of the world.

#### Blood type compatibility

The donor and recipient blood type should be compatible. In most of the allocation algorithms, ABO blood type identical transplants are prioritized over compatible transplants. However blood type identical transplants are not prioritized, when there is an emergency patient, or there is a patient with Zero HLA mismatches. UNOS and Euro-transplant have similar rules for ABO-B donors and recipients [47].

#### HLA (human leukocyte antigen) matching

There are antigens in the human tissue cells that vary from person to person. The system of these antigens is called the HLA system [47]. HLA-A, B and DR are significant in kidney transplantation. Each person has up to two different alleles associated with each of the above three antigens [48]. Therefore, the range of mismatch can range from 0 to 0-0 (exact match in all three HLA pairs) to 2–2-2 (all allele pairs are different). The degree of mismatch has an impact on graft survival rates [49].

#### PRA (panel reactive antibodies)

The PRA value indicates the level of sensitivity of a patient to human leukocyte antigens. The probability of finding a cross-match negative for patients with high PRA values is very low, so these patients may wait a long time to receive a compatible kidney or never find a compatible kidney. To have an equitable system, these patients are prioritized above those with low PRA values [50, 51]. Based on the opinion of the experts who contributed in this research and some kidney allocation models [52], PRA > 80% were considered for the determination of patients with high sensitivity.

#### Age difference

Most sources point out that the kidney from an elderly donor should not be allocated to a very young patient [11]. Age difference between the donor and the recipient is one of the important factors in the graft survival.

#### Waiting time

One of the criteria that is considered in most of allocation algorithms for respecting equity between patients is the length of time that a patient is on a waiting list. There are two approaches to calculating the waiting time. Some of the allocation algorithms are considered when the patient registers in the waiting list and is in active state [53], but others are considered when the patient starts an alternative treatment; e.g. he/she starts dialysis [52, 54].

#### Medical urgency

If a patient has a very urgent condition and vascular access to dialysis is not feasible, the only treatment method is transplantation. Such patients would have high priority to transplant. In some kidney allocation models such as ETKAS are considered several levels for medical urgency [55], but in some models such as kidney allocation models in Australia [52] and Iran, the medical urgency is defined as a two-state variable (whether a patient is an urgency or not). In this research, we consider the medical urgency as a two-state variables.

#### Location

Distance between the transplant center (TC) and the organ procurement unit (OPU) is one of the criteria that is considered in some allocation algorithms [52–55].

#### Transplant status (first time vs. repeat)

Some allocation algorithms make a difference between the patients requiring re-transplant and the patients to be transplanted for the first time.

#### A prior living donor

Some systems give bonus for transplant candidates having donated one of their own kidneys. The bonus of these patients can encourage people to donate. UNOS and Euro-transplant also give bonus for this factor [54, 55].

#### Recipient age

Almost in all allocation systems, special attention is given to pediatric recipients. The age range that is considered in different systems is different. For example, in UNOS, patients under 11 years old and patients aged 11–18 years receive different points [54]. In Euro-transplant, patients under 6 years old, patients aged 6–11 years, and patient aged 11–16 years receive 100, 66.6 and 33.3 points, respectively [55]. In Iran, patients under 18 years old have priority.

#### Predicted survival

To have an efficient allocation, in addition to the criteria listed above, many other characteristics of donor and recipient that can affect the transplant outcomes and graft survival such as a history of diabetes, blood pressure, cardiovascular diseases (CVDs), gender or even the cause of brain death should be considered [56–59]. In many countries, these factors are not considered to simplify the allocation algorithm. A lot of research has been done to predict the graft survival with the characteristics of donor and recipient [60–64] . In this study, the criterion of predicted survival is considered assuming that graft survival can be predicted.

### Step 2: weighing the criteria with IF-AHP

AHP is a popular decision making tool, which was first introduced by Saaty [65]. It is a method for decision-making, aiming at solving complex problems. AHP helps decision makers in making a right decision and better understanding of the problem [66]. Since decision makers may be faced with doubt and uncertainty when comparing the criteria, therefore, to consider their uncertainty in formulating the problem, in this study, IF-AHP has been used. The introduction of Intuitionistic Fuzzy Sets (IFS) is presented in Additional file 3: Appendix A.

The steps of IF-AHP are as follows:

1. Building analytical hierarchy framework

**Fig. 3:**
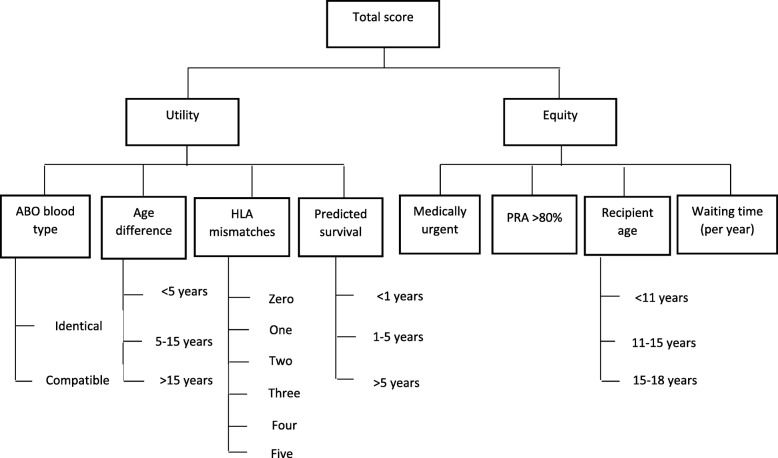
The AHP framework at all levels

Define objective, criteria, and sub-criteria, and then build the hierarchy framework. The hierarchical framework is shown in Fig. 3.

Between the organ allocation experts and in the literature of this field, there are different views on the relation of the sub-criteria to the top-level criteria (utility and equity). Several of the sub-criteria do not fit neatly under either the utility or equity. For example, in the case of the recipient age, some of researchers believes that prioritizing younger recipient is related to utility, in term of maximizing the value and benefit of receiving an organ as well as increased graft longevity. Undeniably life expectancy for older transplant recipients is shorter than that for younger patients [67]. But, others believe that prioritizing younger recipient is related to equity. They argue that prioritizing younger patients is more.

equitable, not because they are likely to benefit most, rather because they have not yet had the opportunity to reach the same point in life as the older patient [68]. There are similar discussions in literature about medical urgency, PRA, and waiting time.

2. Construct pairwise comparison matrices

Construct pairwise comparison matrices for criteria and sub-criteria, and collect the experts’ judgments using Triangular Intuitionistic Fuzzy (TIF) scale. We applied the 5-point scale used in Otay et al.’s research (Table 2, [32] to simplify the questionnaire, but a 7-point scale or 9-point scale can also be used.

**Table 2:** Scale for pairwise comparisons

Importance level	Corresponding intuitionistic fuzzy sets	Reciprocal intuitionistic fuzzy sets
Equal Importance (EI)	(1, 1, 1; 1, 1, 1)	(**1.1.1**; **1.1.1** )
Weak Importance (WI)	(2, 3, 4; 1, 3, 5)	$\left(\frac{\mathbf{1}}{\mathbf{4}}.\frac{\mathbf{1}}{\mathbf{3}}.\frac{\mathbf{1}}{\mathbf{2}};\frac{\mathbf{1}}{\mathbf{5}}.\frac{\mathbf{1}}{\mathbf{3}}.\mathbf{1}\ \right)$
Fairly Strong Importance (FSI)	(4, 5, 6; 3, 5, 7)	$\left(\frac{\mathbf{1}}{\mathbf{6}}.\frac{\mathbf{1}}{\mathbf{5}}.\frac{\mathbf{1}}{\mathbf{4}};\frac{\mathbf{1}}{\mathbf{7}}.\frac{\mathbf{1}}{\mathbf{5}}.\frac{\mathbf{1}}{\mathbf{3}}\ \right)$
Very Strong Importance (VSI)	(6, 7, 8; 5, 7, 9)	$\left(\frac{\mathbf{1}}{\mathbf{8}}.\frac{\mathbf{1}}{\mathbf{7}}.\frac{\mathbf{1}}{\mathbf{6}};\frac{\mathbf{1}}{\mathbf{9}}.\frac{\mathbf{1}}{\mathbf{7}}.\frac{\mathbf{1}}{\mathbf{5}}\ \right)$
Absolute Importance (AI)	(8, 9, 9; 7, 9, 9)	$\left(\frac{\mathbf{1}}{\mathbf{9}}.\frac{\mathbf{1}}{\mathbf{9}}.\frac{\mathbf{1}}{\mathbf{8}};\frac{\mathbf{1}}{\mathbf{9}}.\frac{\mathbf{1}}{\mathbf{9}}.\frac{\mathbf{1}}{\mathbf{7}}\ \right)$

A Triangular intuitionistic fuzzy number (TIFN) is defined with the following membership and non-membership functions, respectively:

3 $$ {\mu}_{\overset{\sim }{A\ }}(x)=\left\{\begin{array}{c}\frac{\mathrm{x}-{a}^L}{a^M-{a}^L}\ .\mathrm{for}\ {a}^L\le \mathrm{x}\le {a}^M\\ {}\frac{a^U-\mathrm{x}}{a^U-{a}^M}\ .\mathrm{for}\ {a}^M\le \mathrm{x}\le {a}^U\\ {}0. otherwise\end{array}\right. $$

and

4 $$ {v}_{\overset{\sim }{A\ }}(x)=\left\{\begin{array}{c}\frac{a^M-x}{a^M-{{a}}^L}\ .\mathrm{for}\ {{a}}^L\le \mathrm{x}\le {a}^M\\ {}\frac{x-{a}^M}{{{a}}^U-{a}^M}\ .\mathrm{for}\ {a}^M\le \mathrm{x}\le {{a}}^U\\ {}\ \\ {}\ 1. otherwise\end{array}\right. $$

$$ where\ {{a}}^L\le {a}^L\le {a}^M\le {a}^U\le {{a}}^U. and\ 0\le {\mu}_{\overset{\sim }{A\ }}(x)+{v}_{\overset{\sim }{A\ }}(x)\le 1. for\ every\  x\epsilon X $$

$$ TIFN\ is\ donated\  by\ {\overset{\sim }{A}}_{TIFN}=\left(\ {a}^L.{a}^M.{a}^U;{{a}}^L.{a}^M.{{a}}^U\right) $$

The pairwise evaluation matrix of each expert is as follows:

5 $$ {\overset{\sim }{A}}^{TIFN}=\left[\begin{array}{cccc}\left(\mathrm{1.1.1};\mathrm{1.1.1}\right)& {\overset{\sim }{a}}_{12}^{TIFN}& \dots & {\overset{\sim }{a}}_{1t}^{TIFN}\\ {}1/{\overset{\sim }{a}}_{12}^{TIFN}& \left(\mathrm{1.1.1};\mathrm{1.1.1}\right)& \dots & {\overset{\sim }{a}}_{2t}^{TIFN}\\ {}\vdots & \vdots & \ddots & \vdots \\ {}1/{\overset{\sim }{a}}_{1t}^{TIFN}& 1/{\overset{\sim }{a}}_{2t}^{TIFN}& \dots & \left(\mathrm{1.1.1},\mathrm{1.1.1}\right)\end{array}\right] $$

$$ {\overset{\sim }{a}}_{12}^{TIFN}=\left(\ {a}_{12}^L.{a}_{12}^M.{a}_{12.i}^U;{{a}}_{12}^L.{a}_{12}^M.{{a}}_{12}^U\right) $$

and

$$ 1/{\overset{\sim }{\mathrm{a}}}_{12}^{\mathrm{TIFN}}=\left(\ \frac{1}{{\mathrm{a}}_{12}^{\mathrm{U}}}.\frac{1}{{\mathrm{a}}_{12}^{\mathrm{M}}}.\frac{1}{{\mathrm{a}}_{12}^{\mathrm{L}}};\frac{1}{{{\mathrm{a}}}_{12}^{\mathrm{U}}}.\frac{1}{{\mathrm{a}}_{12}^{\mathrm{M}}}.\frac{1}{{{\mathrm{a}}}_{12}^{\mathrm{L}}}\ \right) $$

3. Examine consistency of the fuzzy pairwise comparison matrices

For this purpose, the matrix is defuzzified and its consistency is checked. Using Eq. (6), we checked the consistency of matrices [34]. If CR < 0.1, then the comparisons are acceptable, otherwise, they are not acceptable and the values should be revised [28]. The random index (RI) has been taken from Saaty [69].

6 $$ CR=\frac{\left({\lambda}_{max}-n\right)/\left(n-1\right)}{RI} $$

4. Calculate geometric mean of each row in matrices

The goal of this step is to aggregate the expert opinions in one matrix. For this purpose, the geometric mean of each row in the matrices is calculated using Eqs. (7) and (8):

7 $$ \tilde{g}_{r}={\left[\tilde{a}_{r}1^{TIFN}\otimes \cdots \otimes \tilde{a}_{r}n^{TIFN}\ \right]}^1\!/ n $$

$$ where $$

8 $$ \tilde{g}_{r}^{TIFN}=\left(\genfrac{}{}{0pt}{}{{\left(\prod \limits_{j=1}^n{a}_{rj}^L\ \right)}^{\frac{1}{n}}.{\left(\prod \limits_{j=1}^n{a}_{rj}^M\ \right)}^{\frac{1}{n}}.{\left(\prod \limits_{j=1}^n{a}_{rj}^U\ \right)}^{\frac{1}{n}};}{{\left(\prod \limits_{j=1}^n{{a}}_{rj}^L\ \right)}^{\frac{1}{n}}.{\left(\prod \limits_{j=1}^n{a}_{rj}^M\ \right)}^{\frac{1}{n}}.{\left(\prod \limits_{j=1}^n{{a}}_{rj}^U\ \right)}^{\frac{1}{n}}}\right) $$

5. Calculate triangular intuitionistic fuzzy weights

The weight of each criterion and sub-criterion is calculated using Eq. (9):

9 $$ \tilde{w}_{r}^{TIFN}=\tilde{g}_{r}^{TIFN}\otimes {\left[{\overset{\sim }{g}}_1^{TIFN}\oplus \cdots \oplus {\overset{\sim }{g}}_2^{TIFN}\oplus \cdots \oplus \tilde{g}_{t}^{TIFN}\right]}^{-1} $$

6. Defuzzify fuzzy weights to determine importance weights of criteria

For the final ranking, the calculated weights in step 5 should be defuzzified. We use the defuzzification function given in Otay et al. [32] (Eq. 10):

10 $$ {d}_f=\frac{a_i^L+{a}_i^M+{a}_i^U}{3}+\frac{{{a}}_i^L+{a}_i^M+{{a}}_i^U}{\tau } $$

Where, τ is a very large number. It is the non-membership impact factor; as it gets larger, the effect of non-membership function in defuzzification gets smaller. Its value is determined by decision makers according to the type of problem.

## Results

### Identification of essential factor using FDM

From the 11 factors gathered from the literature and different allocation systems, eight essential factors were identified, and three factors (location, a prior donation and transplant status) were rejected by the experts. FDM calculation is presented in Table 3. The reason for the experts to reject the location factor was that since in the current system of kidney allocation in Iran, instead of transporting the kidneys from one city to another, the patients are transported from their place of residence to the city where they are to be transplanted there, so there is no need to consider “location” as an essential factor. They believe that transporting of the recipient than the kidney leads to better results. In the case of a prior donation, since it is allowed to buy and sell kidneys in Iran, therefore, considering this factor as a point for patients is not necessary. In Iran, re-transplanted patients are treated similar to ordinary patients; hence, the transplant status was rejected. About the predicted survival factor, consensus was on considering this factor as one of the essential factors in the kidney allocation. Although this factor was accepted, there was concern that because many interfering factors are needed to predict the graft survival, and practically these factors are less well recorded and followed in Iran, therefore, we will have difficulty in calculating this factor.

**Table 3:** FDM results on selection/rejection of kidney allocation factors

Factors	Fuzzy weights	Defuzzy weights	Selected/ Rejected
Blood type compatibility	(0.3,0.6642671, 1)	0.654756	Selected
HLA matching	(0.3, 0.784858, 1)	0.694953	Selected
PRA	(0.5, 0.904495, 1)	0.801498	Selected
Age difference	(0.5, 0.814531, 1)	0.77151	Selected
Recipient Age	(0.5, 0.844001, 1)	0.781334	Selected
Location	(0, 0.252012, 0.9)	0.384004	Rejected
Transplant status	(0, 0.405595, 0.9)	0.435198	Rejected
Waiting time	(0.3, 0.903077, 1)	0.734359	Selected
Medical urgency	(0.3, 0.898851, 1)	0.73295	Selected
Predicted survival	(0.3, 0.779734, 1)	0.693245	Selected
A prior living donor	(0, 0.424355, 1)	0.474785	Rejected
THRESHOLD	(0.27272, 0.69779, 0.98181)	0.650781	

### Obtaining the weight of criteria and sub-criteria using IF-AHP method

The results of IF-AHP analysis are shown in Tables 4, 5, 6 and 7. Tables 4, 5 and 6 show the pairwise comparison of criteria and sub-criteria obtained from the experts’ judgments. Table 7 presents the weights obtained for criteria and sub-criteria including relative weights and global weights. The global weights were obtained by multiplying the relative weights of the criteria with the relative weights of sub-criteria [42]. The final ranking is based on the global weight values.

**Table 4:** Pair-wise comparison of criteria

	Equity	Utility
Equity	(1,1,1; 1,1,1)	(0.42, 0.50,0.62; 0.37, 0.49, 0.90)
Utility	(1.62, 2.03,2.39; 1.12, 2.03, 2.72)	(1,1,1; 1,1,1)

**Table 5:** Pair-wise comparison of sub-criteria for equity

	Medical Urgency	PRA	Recipient Age	Waiting Time
M-U	(1,1,1; 1,1,1)	(3.78,4.36,4.82;3.16,4.36,5.24)	(2.3,2.52,2.66;2.05,2.52,2.78)	(6.21,7.24,7.92;5.16,7.24,8.56)
PRA	(0.21,0.23,0.26;0.19,0.23,0.32)	(1,1,1; 1,1,1)	(0.46,0.49,053;0.44,0.49,0.58)	(2.3,2.72,3.14;1.87,2.72,3.59)
R-A	(0.38,0.4044;0.36,0.4,0.49)	(1.89,2.04,2.17;1.72,2.04,2.29)	(1,1,1; 1,1,1)	(4.7,5.72,6.73;3.68,5.72,7.74)
W-T	(0.13,0.14,0.16;0.12,0.14,0.19)	(0.32,0.37,0.44;0.28,0.37,0.53)	(0.15,0.17,0.21;0.13,0.17,0.27)	(1,1,1; 1,1,1)
	CR = 0.023 < 0.1

**Table 6:** Pair-wise comparison of sub-criteria for utility

	HLA matching	ABO	Age Difference	Survival Predicted
HLA	(1,1,1; 1,1,1)	(1.34,1.43,1.52;1.24,1.43,1.6)	(3.23,3.65,4.05;2.79,3.65,4.43)	(0.96,1.17,1.45;0.79,1.17,1.95)
ABO	(0.66,0.7,0.75;0.62,0.7,0.81)	(1,1,1; 1,1,1)	(1.52,1.68,1.83;1.34,1.68,2)	(0.21,0.25,0.31;0.18,0.25,0.41)
A-D	(0.25,0.27,0.31;0.23,0.27,0.36)	(0.55,0.6,0.66;0.5,0.6,0.74)	(1,1,1;1,1,1)	(0.33,0.37,0.42;0.3,0.37,0.5)
S-P	(0.7,0.86,1.04;0.51,0.86,1.27)	(3.2,3.94,4.7;2.43,3.94,5.52)	(2.4,2.72,3.02;2.03,2.72,3.3)	(1,1,1;1,1,1)
	CR = 0.06272 < 0.1

**Table 7:** Local and global weights of all criteria and sub-criteria using IFAHP method

Criteria	Relative weights using IFAHP	Sub-criteria	Relative weights using IFAHP	Rank	Global weights using IFAHP	Rank
Equity	0.33	Medical urgency	0.54	**1**	0.1782	1
		PRA	0.14	3	0.0462	9
		Recipient age	0.27	2		
		- < 11 years (0.54)			0.048114	8
		- 11–15 years (0.29)			0.025839	10
		- 15–18 years(0.16)			0.014256	16
		Waiting time^a^	0.05	4	0.0165	14
Utility	0.67	HLA matching	0.35	2		
		- 0 mismatches(0.56)			0.13132	3
		- 1 mismatch (0.21)			0.049245	7
		- 2 mismatches (0.11)			0.025795	11
		- 3 mismatches (0.06)			0.01407	17
		- 4 mismatches (0.04)			0.00938	18
		- 5 mismatches (0.02)			0.00469	20
		Blood type compatibility	0.16	3		
		- Identical (0.83)			0.088976	4
		- Compatible (0.17)			0.018224	12
		Age difference	0.11	4		
		- < 5 years (0.69)			0.050853	6
		- 5–15 years (0.24)			0.017688	13
		- > 15 years (0.07)			0.005159	19
		Predicted survival	0.38	1		
		- < 1 years (0.06)			0.015276	15
		- 1–5 years (0.26)			0.066196	5
		- > 5 years (0.68)			0.173128	2

^a^Calculated weight for waiting time is for one year

The results of Table 7 show that the “medical urgency” received the highest weight, and the “5 HLA mismatches” received the lowest weight. These results are similar to those of the ETKAS algorithm. In ETKAS, the highest point is assigned to high urgency patients (500 points) and the lowest point is assigned to 5 HLA mismatches (33.3 points) [55]. Table 10 shows the factors with the highest and lowest weights in different allocation algorithms.

### Model evaluation

The developed model was evaluated in two steps. In the first step, the model was implemented using available data, which was the kidney transplantation dataset in Tehran (one of the 15 regions that has longest waiting list) from October 2017 to December 2017. This dataset included 484 registered patients and 124 deceased donors. The proposed model was run for each donated kidney and the chosen patients of each run were recorded. Chosen patients of existing system were available. Results of the existing model and proposed one have been compared. Given that there was no factor indicating the success of transplantation in the dataset, we used EPTS score to compare the utility of the models. Considering that EPST score can only be calculated for the patients over the age of 18 years; therefore, this criterion was calculated only for adult patients. The results are shown in Table 8.

**Table 8:** Comparing the results of the developed model and the current allocation model in Iran

Measures	Recipient type	Developed model	Current model
Measures of utility			
The number of recipients with an EPTS < 20%	Adult	124 of 228 (54%)	83 of 230 (36%)
Average EPTS score of recipients(%)	Adult	24.61%	41.37%
Average age difference between donors and recipients	All	5.3 years	8.1 years
The number of allocation with the identical blood type	All	243 of 248 (98%)	248 of 248 (100%)
Measures of equity			
Average waiting time of all recipients	All	1.25 years	1.7 years
Average waiting time of urgency patients	Urgency	1.1 years	0.9 years
The number of recipients< 18 y out of patients< 18 y waiting	Pediatric	20 of 22	18 of 22

Based on the results presented in Table 8, the developed method is capable to improve the measures of utility. The number of recipients with an EPTS < 20% has increased from 83 to 124 (18%), and the average age difference between the donor and recipient has decreased from 8.1 years to 5.3 years. Only the number of allocations with the identical blood type in the existing system is better than that in the proposed system. This is due to the fact that in the existing system, the allocation is based on the identical blood type. Also, in the case of measures of equity, the proposed model is better than the current model. Although the most important allocation factor in the current model is waiting time, it can be seen that the proposed model has decreased the average waiting time of all recipients from 1.7 years to 1.25 years. Large waiting time reductions are only possible with a large increase in the number of available donors or a reduction in demand for organs. Maybe waiting time reduction in proposed model is caused because the limitation of the identical blood type for allocation has been considered in the existing model, while it has been eliminated in the proposed model. In the case of urgency patients, the existing system works better. It is true that medical urgency is the most important factor in the proposed model, but the allocation is based on the total score of all factors. In the existing system, emergency patients will receive the highest priority if the rest of the factors are compatible. Therefore, it is logical that the existing model has a shorter waiting time for emergency patients.

**Fig. 4:**
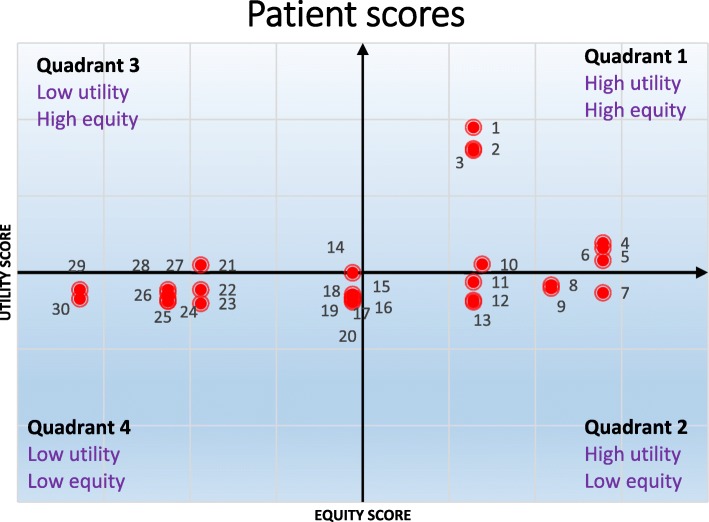
Patients’ scores plot of utility versus equity

In the second step, to confirm the proposed model by experts and decision makers, a sample of 30 was selected from the dataset and the patients were prioritized for receiving a donated kidney with both models (current and proposed models). The high priorities of the proposed model were (**7**, 15, 1, 4, 8, 12) and the high priorities of the current model were (8, 10, 4, 12, 15, 2). The total score of the patient is equal to the sum of utility score and equity score. Patient scores are summarized in Table 9. Figure 4 shows the profile matrix in which the patient’s location is determined by the utility(X-axis) and the equity score(Y-axis). This type of display helps decision makers to more easily compare the results of the proposed and current models. The means of the utility score and equity score are subtracted from each patient’s utility score and equity score to generate the X and Y-coordinates for the plot [26].

**Table 9:** Patients’ scores and the ranks in proposed model for all the 30 patients

Patient Id	Patients’ factors				Utility score	Equity score	Total score	Patient Rank (proposed model)
	M-U	ABO	WT	Age				
1	Yes	O	23	74	0.293	0.255	0.548	3
2	No	O	5	8	0.338	0.079	0.417	8
3	No	O	7	38	0.135	0.055	0.190	23
4	No	O	30	7	0.368	0.134	0.502	4
5	No	O	44	34	0.135	0.105	0.240	21
6	No	O	20	44	0.135	0.073	0.208	22
7	Yes	O	46	35	0.293	0.285	0.578	1
8	No	O	25	48	0.368	0.127	0.495	5
9	No	O	28	24	0.293	0.083	0.376	11
10	No	O	2	35	0.338	0.075	0.413	9
11	No	O	17	32	0.368	0.069	0.437	7
12	No	O	13	58	0.368	0.111	0.479	6
13	No	O	10	9	0.293	0.059	0.352	12
14	No	O	8	36	0.293	0.057	0.350	13
15	Yes	O	5	15	0.293	0.257	0.550	2
16	No	A	1	34	0.223	0.095	0.318	14
17	No	AB	20	51	0.116	0.073	0.189	24
18	No	A	20	58	0.065	0.073	0.138	29
19	No	B	17	34	0.116	0.069	0.185	25
20	No	B	16	14	0.223	0.067	0.290	15
21	No	AB	13	6	0.223	0.063	0.286	16
22	No	A	13	38	0.223	0.063	0.286	17
23	No	A	11	46	0.116	0.061	0.177	26
24	No	A	11	34	0.065	0.061	0.126	30
25	No	B	11	56	0.223	0.061	0.284	18
26	No	AB	10	55	0.223	0.059	0.282	19
27	No	B	9	54	0.116	0.058	0.174	27
28	No	A	9	13	0.298	0.106	0.404	10
29	No	A	9	3	0.116	0.058	0.174	28
30	No	AB	8	39	0.223	0.057	0.280	20

As shown in Fig. 4, all six patients selected by the developed method (**7**, 15, 1, 4, 8, 12) are in the high priority patients’ quadrant while only four patients selected by the current model (8, 4, 12, 15) are in this quadrant. The allocation trend of the proposed model is shown in Fig. 4. As can be seen, the patients in quadrant 1 are selected first, and then the patients in quadrants 2, 3 and 4 are selected orderly.

### Model sensitivity analysis

The robustness of the proposed method can be assessed by sensitivity analysis. By sensitivity analysis, we can understand the effect of variations in the weight of criteria on patient rankings [26]. Ideally, the model results with minor variations should be stable. A series of sensitivity analysis were conducted to analyze the effect of varying the weight of the criteria on the patients’ ranking. Sensitivity analysis can be performed by changing the weight at all levels, but we only performed levels 1 and 2. The results showed that changes in the main criteria (utility and equity) up to 50% had no effect on the patients’ priority.

In the case of sub-criteria in the second level, multi-scenarios are performed. The sub-criteria were increased and decreased for up to approximately 30%. The results revealed that change in the sub-criteria up to 30% has no significant influence on the patients’ ranking. This indicates that the proposed model is stable and robust, and decision makers can be sure that the selected patients are the most suitable choices.

## Discussion

Kidney transplantation is an appropriate and effective treatment for end-stage renal disease (ESRD). One of the most important challenges in kidney transplantation is the policy of kidney allocation and finding the most suitable recipient. Currently, most of kidney allocation policies are based on making balance between equity and utility so as to consider ethical issues in addition to medical criteria. Leading organizations in organ transplantation such as UNOS and Euro-transplant use a point scoring system to allocate organs. In these systems, different points are considered for each criterion. The donated kidney is allocated to the patient who earns the highest score. The most important challenge in designing these systems is identifying effective factors and weighting them to get the best utility and equity outcomes. Therefore, the main objective of this study was to provide a framework for identification of the essential criteria for the kidney allocation and weighting them. The research was carried out in two steps. In the first step, after extracting the criteria from the literature and different allocation models, FDM was used to identify the essential criteria. Of the 11 factors (10 factors extracted from the literature plus the predicted survival factor that we proposed), eight factors were confirmed.

In the second step, the hierarchical structure was drawn in four levels, and using IF-AHP method, the weighted criteria and sub-criteria were determined. In this research, triangular intuitionistic fuzzy numbers were used instead of crisp numbers or fuzzy numbers in the AHP model to consider the skeptics and uncertainty of the decision makers in pairwise comparisons of the criteria. This paper is the first to use this approach to determine the weight of the criteria in an organ allocation algorithm. The results showed that, the 5 HLA mismatches should have the lowest weight, and medical urgency should have the highest weight. These results are similar to those of the ETKAS algorithm used for kidney allocation in Euro-transplant. Table 10 shows the factors with the highest and lowest weights in different allocation algorithms.

**Table 10:** Highest and lowest points in several allocation algorithms

Country (Algorithm)	Highest point	Lowest point	Source
Euro-transplant (ETKAS)	High Urgency, simultaneous liver-kidney transplant, a prior living donor (500 points)	Waiting time, 5 HLA mismatches, Recipient age 11–16 years (33.3 points)	[55]
Australia	Zero HLA mismatches and peak PRA > 50% (60,000,000 points)	Waiting time (1200 points)	[52]
New Zealand	DR HLA mismatches (2200 point in Rank 1, 300 points in Rank 2)	Waiting time (12 points in Rank 1, 36 points in Rank 2)	[52]
Turkey (TONKS)	Zero HLA mismatches (7 points)	3 HLA mismatches (0 points)	[12]
Proposed model	Medical Urgency (global weight = 0.1782)	5 HLA mismatches (global weight = 0.0046)	

US kidney allocation system (KAS) was not included since it is not based strictly on a points system, but rather is a classification-driven system with points playing a secondary role. In KAS, patients are first ordered by classification, and points are only used to further sort patients within classification. KAS awards very high priority for zero HLA mismatch, highly sensitized patients (CPRA 98–100%), prior living donors, and pediatric patients [54]

To evaluate the developed method, firstly, it was implemented using a real case study from the Iranian Kidney Allocation System. The results showed that the proposed model has the potential to improve the allocation outcomes. Secondly, to confirm the proposed model by decision makers, the selected patients by the developed model and the current model were graphically displayed in a profile matrix in which the patient’s location is determined by the utility score (X-axis) and equity score (Y-axis). The allocation trend of the proposed model indicated that it allocates better than the current system. Finally, sensitivity analysis was applied to approve the robustness of the model by using several what-if scenarios.

## Conclusions

To the best of our knowledge, this is a first study, which integrates FDM with IF_AHP together in developing organ allocation system. FDM was used to identify the essential criteria, and IF_AHP was used to determine the weight of them by considering the skeptics and uncertainty of the decision makers in pairwise comparisons of the criteria. The framework presented in this study is suitable for the countries that intend to improve their organ allocation systems.

It is suggested that future research employ the allocation model for other organs with the same approach; weighing the criteria be done with three different methods (ordinary AHP, fuzzy AHP and IF-AHP), and the results are compared. In this paper, the kidney allocation criteria were identified and weighted; however, developing a kidney allocation system was out of this work’s scope. So the authors are planning to develop an intuitionistic fuzzy expert system in order to replace allocator in the decision making in their future research works.

## Additional files

Additional file 1: Questionnaire 1. The questionnaire used in the research to identify the essential factors in kidney allocation by fuzzy Delphi method. (DOCX 18 kb)

Additional file 2: Questionnaire 2. The questionnaire used in the research to weigh the factors by Intuitionistic Fuzzy Analytic Hierarchy Process (IF-AHP) method. (DOCX 22 kb)

Additional file 3: Appendix A. The introduction of Intuitionistic Fuzzy Sets. (DOCX 65 kb)

## Data Availability

The datasets used during this study are available from the corresponding author on reasonable request.

## Abbreviations

EPTS: Estimated Post Transplant Survival
ETKAS: Euro-transplant kidney allocation system
FDM: Fuzzy Delphi Method
HLA: Human Leukocyte Antigen
IF_AHP: Intuitionistic Fuzzy Analytic Hierarchy Process
IFS: Intuitionistic fuzzy set
PRA: Panel Reactive Antibodies
UNOS: United Network for Organ Sharing
