# Supplementary material for: Identification and weighting of kidney allocation criteria: a novel multi-expert fuzzy method
Source: BMC Med Inform Decis Mak. 2019 Sep 6;19:182. doi: 10.1186/s12911-019-0892-y (PMC6729045; doi:10.1186/s12911-019-0892-y)
Supplement: Supplementary file 1 — Questionnaire 1. The questionnaire used in the research to identify the essential factors in kidney allocation by fuzzy Delphi method. (DOCX 18 kb) [file 12911_2019_892_MOESM1_ESM.docx]

Dear Sir/Madam,

The current questionnaire is designed to identify the factors affecting kidney allocation in Iran.

Your involvement and accuracy in answering questions will certainly be effective in the success of the questionnaire and therefore of our research. The answers you provide on the questionnaire will be kept strictly confidential and no data will be shared about the individual and will not be used for any purpose other than our research.

We will be pleased to share with you the findings we obtained as a result of our research in the relevant area. Thank you very much for your involvement.

Best Regards,

Nasrin Taherkhani

e-mail: [taherkhani.n@gmail.com](mailto:taherkhani.n@gmail.com)

**Please determine the importance of each factor for present of a kidney allocation system.**

**Example**

While evaluating the "Waiting Time" in the first line, if you mark "VH", you mention that the importance of the presence of this factor in the kidney allocation system is "very high"

**VH=Very High H=High MH=Medium High M=Medium**

**ML=Medium Low L=Low VL=Very Low**

| **Factors** | **VH** | **H** | **MH** | **M** | **ML** | **L** | **VL** |
| --- | --- | --- | --- | --- | --- | --- | --- |
| Waiting Time |  |  |  |  |  |  |  |
| PRA |  |  |  |  |  |  |  |
| Pediatric patients (less than18 years) |  |  |  |  |  |  |  |
| Identical blood type vs Compatible blood type |  |  |  |  |  |  |  |
| A prior living donor |  |  |  |  |  |  |  |
| HLA matching |  |  |  |  |  |  |  |
| Medical urgency |  |  |  |  |  |  |  |
| Location |  |  |  |  |  |  |  |
| Transplant status |  |  |  |  |  |  |  |
| Age difference |  |  |  |  |  |  |  |
| Predicted survival |  |  |  |  |  |  |  |
