# Supplementary material for: Identification and weighting of kidney allocation criteria: a novel multi-expert fuzzy method
Source: BMC Med Inform Decis Mak. 2019 Sep 6;19:182. doi: 10.1186/s12911-019-0892-y (PMC6729045; doi:10.1186/s12911-019-0892-y)
Supplement: Supplementary file 2 — Questionnaire 2. The questionnaire used in the research to weigh the factors by Intuitionistic Fuzzy Analytic Hierarchy Process (IF-AHP) method. (DOCX 22 kb) [file 12911_2019_892_MOESM2_ESM.docx]

Dear Sir/Madam,

The current questionnaire is designed to weigh the factors affecting kidney allocation in Iran.

Your involvement and accuracy in answering questions will certainly be effective in the success of the questionnaire and therefore of our research. The answers you provide on the questionnaire will be kept strictly confidential and no data will be shared about the individual and will not be used for any purpose other than our research.

We will be pleased to share with you the findings we obtained as a result of our research in the relevant area. Thank you very much for your involvement.

Best Regards,

Nasrin Taherkhani

e-mail: [taherkhani.n@gmail.com](mailto:taherkhani.n@gmail.com)

**Please judge the relative importance: How important is each element on the left (A) compared with each element on the right (B)?**

**Example**

While evaluating the "Equity" and "Utility" in the first line, if you mark "AI" on the "Equity " side, you would prefer to consider "Equity " as " Absolute Importance " rather than "Utility". Similarly, if you think that "Equity" are equal to "Utility", you should mark “E” in the middle.

**AI=Absolute Importance VSI=Very Strong Importance FSI=Fairly Strong Importance**

**WI=Weak Importance EI=Equal Importance**

| **Equity vs Utility** | | | | | | | | | | |
| --- | --- | --- | --- | --- | --- | --- | --- | --- | --- | --- |
|  | AI | VSI | FSI | WI | EI | WI | FSI | VSI | AI |  |
| Equity |  |  |  |  |  |  |  |  |  | Utility |
| **Comparison of Equity criteria** | | | | | | | | | | |
|  | AI | VSI | FSI | WI | EI | WI | FSI | VSI | AI |  |
| Medical Urgency |  |  |  |  |  |  |  |  |  | Waiting Time |
| Medical Urgency |  |  |  |  |  |  |  |  |  | PRA>80% |
| Medical Urgency |  |  |  |  |  |  |  |  |  | Pediatric patient |
| PRA>80% |  |  |  |  |  |  |  |  |  | Waiting Time |
| PRA>80% |  |  |  |  |  |  |  |  |  | Pediatric patient |
| Pediatric patient |  |  |  |  |  |  |  |  |  | Waiting Time |
| **Comparison of Recipient age sub criteria** | | | | | | | | | | |
|  | AI | VSI | FSI | WI | EI | WI | FSI | VSI | AI |  |
| Age < 11 years |  |  |  |  |  |  |  |  |  | Age 11 - 15 years |
| Age < 11 years |  |  |  |  |  |  |  |  |  | Age 15 – 18 years |
| Age 11-15 years |  |  |  |  |  |  |  |  |  | Age 15-18 years |
| **Comparison of Equity criteria** | | | | | | | | | | |
|  | AI | VSI | FSI | WI | EI | WI | FSI | VSI | AI |  |
| Survival predicted |  |  |  |  |  |  |  |  |  | HLA matching |
| Survival predicted |  |  |  |  |  |  |  |  |  | Age difference |
| Survival predicted |  |  |  |  |  |  |  |  |  | Identical blood type |
| HLA matching |  |  |  |  |  |  |  |  |  | Age difference |
| HLA matching |  |  |  |  |  |  |  |  |  | Identical blood type |
| Age difference |  |  |  |  |  |  |  |  |  | Identical blood type |
| **Comparison of Survival predicted sub criteria** | | | | | | | | | | |
|  | AI | VSI | FSI | WI | EI | WI | FSI | VSI | AI |  |
| Survival < 1 years |  |  |  |  |  |  |  |  |  | Survival 1-5 years |
| Survival < 1 years |  |  |  |  |  |  |  |  |  | Survival > 5 years |
| Survival 1-5 years |  |  |  |  |  |  |  |  |  | Survival > 5 years |
| **Comparison of HLA matching sub criteria** | | | | | | | | | | |
|  | AI | VSI | FSI | WI | EI | WI | FSI | VSI | AI |  |
| Zero mismatches |  |  |  |  |  |  |  |  |  | One mismatch |
| Zero mismatches |  |  |  |  |  |  |  |  |  | Two mismatches |
| Zero mismatches |  |  |  |  |  |  |  |  |  | Three mismatches |
| Zero mismatches |  |  |  |  |  |  |  |  |  | Four mismatches |
| Zero mismatches |  |  |  |  |  |  |  |  |  | Five mismatches |
| One mismatch |  |  |  |  |  |  |  |  |  | Two mismatches |
| One mismatch |  |  |  |  |  |  |  |  |  | Three mismatches |
| One mismatch |  |  |  |  |  |  |  |  |  | Four mismatches |
| One mismatch |  |  |  |  |  |  |  |  |  | Five mismatches |
| Two mismatches |  |  |  |  |  |  |  |  |  | Three mismatches |
| Two mismatches |  |  |  |  |  |  |  |  |  | Four mismatches |
| Two mismatches |  |  |  |  |  |  |  |  |  | Five mismatches |
| Three mismatches |  |  |  |  |  |  |  |  |  | Four mismatches |
| Three mismatches |  |  |  |  |  |  |  |  |  | Five mismatches |
| Four mismatches |  |  |  |  |  |  |  |  |  | Five mismatches |
| **Comparison of Age difference sub criteria** | | | | | | | | | | |
|  | AI | VSI | FSI | WI | EI | WI | FSI | VSI | AI |  |
| < 5 years |  |  |  |  |  |  |  |  |  | 5 - 15 years |
| < 5 years |  |  |  |  |  |  |  |  |  | > 15 years |
| 5 – 15 years |  |  |  |  |  |  |  |  |  | >15 years |
| **Comparison of ABO blood type sub criteria** | | | | | | | | | | |
|  | AI | VSI | FSI | WI | EI | WI | FSI | VSI | AI |  |
| Identical |  |  |  |  |  |  |  |  |  | Compatible |
