# Supplementary material for: Identification and weighting of kidney allocation criteria: a novel multi-expert fuzzy method
Source: BMC Med Inform Decis Mak. 2019 Sep 6;19:182. doi: 10.1186/s12911-019-0892-y (PMC6729045; doi:10.1186/s12911-019-0892-y)
Supplement: Supplementary file 3 — Appendix A. The introduction of Intuitionistic Fuzzy Sets. (DOCX 65 kb) [file 12911_2019_892_MOESM3_ESM.docx]

**Appendix A**

**Intuitionistic Fuzzy Set (IFS):**

In the fuzzy set theory, the degree of membership of an element is between 0 and 1, and the degree of non-membership of an element equals to 1 minus the degree of membership. However, when a decision maker expresses his/her opinion with fuzzy numbers, there may be some hesitation degree in his/her opinion. In this case, the degree of non-membership cannot be equal to 1 minus the degree of membership. Hence, in order to expand the fuzzy sets, IFS was introduced by Atanassov, which considers the degree of hesitation as 1 minus the sum of membership and non-membership degrees [31].

**Definition 1.** Intuitionistic fuzzy set (IFS)

Let $X\neq\emptyset$ be a given set. An IFS is defined as follows:

$\tilde{A}= \left\{ \left\langle x. \mu_{\tilde{A}} \right. \right.\left( x \right). v_{\tilde{A}} \left( x \right)\left. \right\rangle;x\epsilon X\left. \right\}. where \mu_{\tilde{A}}:X\to\left[ 0.\left. 1 \right] and v_{\tilde{A}}:X\to\left[ 0.\left. 1 \right] \right. \right.$ (1)

$0\leq\mu_{\tilde{A}}\left( x \right)+ v_{\tilde{A}}\left( x \right)\leq1.for every x\epsilon X$ (2)

$$Hesitancy is equal to " 1-(\mu_{\tilde{A}}\left( x \right)+ v_{\tilde{A}}(x))"$$

*Where*$. \mu_{\tilde{A}}\left( x \right)$ and $v_{\tilde{A}}\left( x \right)$ are called the membership and non-membership degrees of x, respectively. The amount of hesitancy about the x is equal to $1-(\mu_{\tilde{A}}\left( x \right)+ v_{\tilde{A}}(x))$. The low amount of hesitancy means having more definitive knowledge about x.

**Definition 2.** Triangular intuitionistic fuzzy numbers(TIFNs):

A TIFN is defined in R with the following membership and non-membership functions, respectively:

$\mu_{\tilde{A}}\left( x \right)=\left\{ \begin{aligned} \frac{x-a^{L}}{a^{M}-a^{L}} &. for a^{L}\leq x\leq a^{M} \\ \frac{a^{U}-x}{a^{U}-a^{M}} &. for a^{M}\leq x\leq a^{U} \\ 0 . otherwise \end{aligned} \right.$ (3)

and

$v_{\tilde{A}}\left( x \right)=\left\{ \begin{aligned} \frac{a^{M}-x}{a^{M}-\acute{a}^{L}} &. for \acute{a}^{L}\leq x\leq a^{M} \\ \frac{x-a^{M}}{\acute{a}^{U}-a^{M}} &. for a^{M}\leq x\leq\acute{a}^{U} \\ \\ 1 . otherwise \end{aligned} \right.$ (4)

$$where \acute{a}^{L}\leq a^{L}\leq a^{M}\leq a^{U}\leq\acute{a}^{U}. and 0\leq\mu_{\tilde{A}}\left( x \right)+ v_{\tilde{A}}\left( x \right)\leq1.for every x\epsilon X$$

$TIFN is donated by \tilde{A}_{TIFN}=\left( a^{L}.a^{M}.a^{U};\acute{a}^{L}.a^{M} .\acute{a}^{U} \right)$ (see Fig. 1)


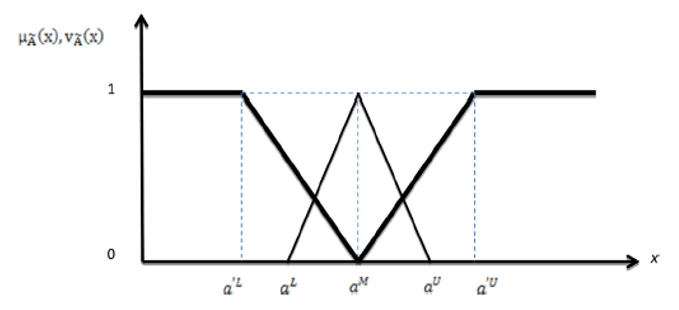


**Fig. 1** Membership and non-membership functions of TIFN

If $\tilde{A}_{TIFN}=\left( a^{L}.a^{M}.a^{U};\acute{a}^{L}.a^{M} .\acute{a}^{U} \right)$ and $\tilde{B}_{TIFN}=\left( b^{L}.b^{M}.b^{U};\acute{b}^{L}.b^{M} .\acute{b}^{U} \right)$ are two TIFNs, then:

**Addition**: $\tilde{C}=\tilde{A}+\tilde{B}$ is also a TIFN:

$\tilde{C}_{TIFN}=\left( {a^{L}+b}^{L}.{a^{M}+b}^{M}.{a^{U}+b}^{U};\acute{a}^{L}+\acute{b}^{L}.{a^{M}+b}^{M} .\acute{a}^{U}+\acute{b}^{U} \right)$ (5)

**Multiplication:** $\tilde{C}\cong\tilde{A}\otimes\tilde{B}$ is also a TIFN:

$\tilde{C}_{TIFN}\cong\left( {a^{L}b}^{L}.{a^{M}b}^{M}.{a^{U}b}^{U};\acute{a}^{L}\acute{b}^{L}.{a^{M}b}^{M} .\acute{a}^{U}\acute{b}^{U} \right)$ (6)

**Division**: $\tilde{C}\cong\tilde{A}⊘\tilde{B}$ is also a TIFN:

$\tilde{C}_{TIFN}\cong\left( {{a^{L}}/{b^{U}}.{a^{M}}/{b^{M}}.{a^{U}}/{b^{L}};}{\acute{a}^{L}}/{\acute{b}^{U}}{\acute{.{a^{M}}/{b^{M}}. {\acute{a}^{U}}/{\acute{b}^{L}}}} \right)$ (7)

**Multiplication with a constant:**

$k\times\tilde{A}_{TIFN}= \left( {k\times a}^{L}.k\times a^{M}.{k\times a}^{U};{k\times\acute{a}}^{L}.{k\times a}^{M} .{k\times\acute{a}}^{U} \right). k>0$ (8)

**Defuzzification of TIFNs:**

Let $I_{i}=(a_{i}^{L}. a_{i}^{M}. a_{i}^{U}; \acute{a}_{i}^{L}. a_{i}^{M}. \acute{a}_{i}^{U} )$ be a TIFN, then we use the defuzzification function given in Otay et al[31]:

$d_{f}= \frac{a_{i}^{L}+ a_{i}^{M}+ a_{i}^{U}}{3}+ \frac{\acute{a}_{i}^{L}+ a_{i}^{M}+ \acute{a}_{i}^{U}}{\tau}$ (9)

Where, $\tau$ is a very large number. It is the non-membership impact factor; as it gets larger, the effect of non-membership function in defuzzification gets smaller. Its value is determined by decision makers according to the type of problem.

**Aggregation operators for TIFNs:**

Let $I_{i}=(a_{i}^{L}. a_{i}^{M}. a_{i}^{U}; \acute{a}_{i}^{L}. a_{i}^{M}. \acute{a}_{i}^{U} )$ be a set of TIFNs, then Eq.(18) can be used to aggregate these numbers:

$f_{m}\left( I_{1}. I_{2}. \ldots. I_{n} \right)=\binom{[1-\prod_{i=1}^{n} \left( 1-a_{i}^{L} \right). 1-\prod_{i=1}^{n} \left( 1-a_{i}^{M} \right). 1-\prod_{i=1}^{n} \left( 1-a_{i}^{U} \right)] .}{[\prod_{i=1}^{n} \left( \acute{a}_{i}^{L} \right). \prod_{i=1}^{n} \left( a_{i}^{M} \right). \prod_{i=1}^{n} \left( \acute{a}_{i}^{U} \right)]}$ (10)
